# Transcranial magnetic stimulation in the assessment of acupuncture effect on exercise‐induced fatigue

**DOI:** 10.1002/brb3.3575

**Published:** 2024-06-12

**Authors:** Linghui Hu, Zhen Wei, Xiaolei Wang, Wei Wu

**Affiliations:** ^1^ School of Exercise and Health Shanghai University of Sport Shanghai China; ^2^ Department of Pain Management Huadong Hospital Affiliated to Fudan University Shanghai China; ^3^ School of Athletic Performance Shanghai University of Sport Shanghai China

**Keywords:** acupuncture, exercise‐induced fatigue, motor evoked potential, transcranial magnetic stimulation

## Abstract

**Background:**

Acupuncture as a traditional Chinese medicine therapy relies on unique theories to alleviate fatigue. The aim of this study is to evaluate the effect of acupuncture on exercise‐induced fatigue utilizing transcranial magnetic stimulation (TMS).

**Methods:**

A total of 20 participants with regular exercise habits were recruited for this study. All participants were randomly assigned to receive either acupuncture or sham acupuncture intervention for exercise‐induced fatigue. TMS and a heart rate monitor were used to measure the amplitude and latency of motor evoked potential (MEP) as well as heart rate every 5 min over a 30‐min period. The blood lactic acid (BLA) levels were measured using Lactate Scout+ at baseline, 0 min, and 30 min after fatigue. Two‐way repeated measures analysis of variance was utilized to compare the differences between the effects of acupuncture method and time. Bonferroni post hoc tests were conducted to compare specific differences. Statistical significance was set at *p* < .05.

**Results:**

Interaction effect was observed between acupuncture method and time effect in terms of amplitude (*F*_(1, 38)_ = 5.40, *p* < .001, *η* ^2^ = 0.12) and latency (*F*_(1, 38)_ = 3.78, *p* = .008, *η* ^2^ = .09) of MEP. The application of acupuncture can promote the recovery of heart rate especially at 30 min (*p* < .05), but which seem insufficient to generate significant difference in BLA (*F*_(1, 38)_ = 0.067, *p* = .797, *η* ^2^ = 0.002).

**Conclusions:**

Acupuncture can promote the increase of MEP amplitude, shorten MEP latency, and restore heart rate. Preliminary findings provide novel insights for individuals with exercise habits to alleviate fatigue and enhance sports performance.

## Graphical abstract

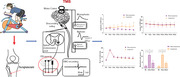

## 1 INTRODUCTION

Exercise‐induced fatigue is a protective response of the body following intense physical training, which may be influenced by various physiological factors, such as energy consumption, lactic acid buildup, and neuroendocrine system dysfunction (Heil et al., 2020; Liu et al., 2022; Pennisi et al., 2019). This fatigue can be categorized into central and peripheral types, impacting sports performance and potentially leading to sports‐related injuries (Chennaoui et al., 2021; Meeusen et al., 2021). Acupuncture as a traditional Chinese therapy has been extensively researched for its potential in alleviating exercise‐induced fatigue during the recovery phase (Yang et al., 2017). Nevertheless, the mechanism of acupuncture to relieve exercise‐induced fatigue remains incompletely understood, particularly its central effects.

Transcranial magnetic stimulation (TMS) is a noninvasive brain stimulation technique that affects the central nervous system (Pennisi et al., 2017; Vernillo et al., 2021) and commonly employed in the diagnosis and treatment of various neurological conditions (Al‐Sultan et al., 2019; Pennisi et al., 2015). Due to its high safety profile, minimal side effects, and clear underlying principles, TMS is frequently utilized as a primary assessment tool for detecting cerebral cortex activity. Given to the fact that motor evoked potential (MEP) can be easily obtained by peripheral muscle electromyography (EMG), an increasing number of studies are investigating the impact of acupuncture on motor cortex excitability (Lo & Cui, 2003; Maioli et al., 2006).

Zusanli (ST 36) is a frequently utilized acupoint in acupuncture practice. Research suggests that acupuncture at ST 36 may effectively decrease subjective fatigue levels, blood lactic acid (BLA), lactate dehydrogenase, heart rate, and other fatigue‐related indicators during physical activity (Jiang et al., 2019). Additionally, it has been reported to possess anti‐inflammatory properties (Oh & Kim, 2021). Sun et al. (2019) also demonstrated that acupuncture at ST 36 can enhance motor cortex excitability at rest, reduce motor cortex inhibition, and have enduring effects.

In relation to these issues, the present study aims to employ TMS technique to explore the impact of needling ST 36 on the motor cortex in the state of exercise‐induced fatigue. Concurrently, heart rate, BLA, and other relevant parameters were observed to establish a theoretical foundation for the use of acupuncture in alleviating exercise‐induced fatigue. We hypothesized that acupuncture may enhance motor cortex excitability and serve as an innovative approach to mitigating exercise‐induced fatigue.

## 2 MATERIALS AND METHODS

A total of 20 sports enthusiasts (get 30 min of exercise at least three times a week) were recruited through the way of poster from the Shanghai University of Sport and the surrounding running groups (20.7 ± 1.6 years, 178.5 ± 10.8 cm, and 70.4 ± 12.6 kg). They were all right‐handed, had no metal implants in their bodies, no history of epilepsy, and refrained from consuming caffeine, alcohol, or drugs within 24 h prior to the commencement of the study (Yu et al., 2020). Prior to participation, all individuals provided written informed consent, and the research protocol was approved by the Ethics Committee of Shanghai University of Sport (NO.102772021RT105)

**FIGURE 1:**
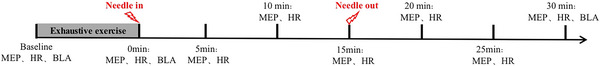
Experimental design. BLA, blood lactic acid; HR, heart rate; MEP, motor evoked potential.

First, the height and body mass of the participants were assessed, thereafter each individual received acupuncture or sham acupuncture intervention immediately after exercise‐induced fatigue randomly with a 1‐week washout interval. All interventions were performed by the same acupuncturist, who was blind with respect to the subject's group allocation and not involved in the subsequent data collection and analysis. The needle was inserted immediately after fatigue and removed 15 min later. MEP and heart rate were collected at baseline, as well as 0‐min, 5‐min, 10‐min, 15‐min, 20‐min, 25‐min, and 30‐min postexercise, respectively. BLA was measured in baseline, after exercise, and after acupuncture intervention (Figure 1).

During the study, muscle fatigue models were developed through the implementation of incremental‐load exhaustive exercise using a bicycle ergometer. A heart rate monitor (Polar H10, Polar Electro Oy) was also used to record participant's heart rate during the experiment. Following adjustment of the seat height for each participant, a warm‐up period at 0 W for 2 min was conducted, after which the initial power was set at 60 W and subsequently increased by 30 W every 2 min. Participants were required to maintain a minimum bicycle ergometer speed of 60 rpm throughout the experiment until they were unable to sustain this speed for more than 5 s (Angius et al., 2019). Meanwhile, the participants' fatigue levels were evaluated immediately using a combination of the rating of perceived exertion (RPE) scale and BLA (Lactate Scout+, EKF).

After confirming the subject's fatigue, the acupuncturist sterilized the ST 36 acupoint using 75% alcohol‐soaked cotton balls. A disposable needle (Hwato, ∅0.30 × 25 mm, Suzhou Medical Appliance Factory) was then inserted vertically, with twisting and pulling motions, until a sunken and tight sensation was felt. For the sham acupuncture procedure, a transparent plastic tube filled with a coupling agent was utilized, following the principles of park sham needles (To & Alexander, 2015). This tube was affixed to ST 36 using double‐sided adhesive tape on one end and stainless‐steel stickers on the other. The needle tip made contact with the sticker without piercing it, providing the subject with the sensation of being needled.

The Ag–AgCl electrodes were positioned at the first dorsal interosseous (FDI) muscle of the right hand, specifically at the metacarpal and phalangeal joints of the index finger and near the styloid process of the ulna to capture surface EMG signals prior to commencing the study. For the TMS (Magstim, Whitland, Dyfed, UK) assessment, which mainly refers to the latest International Federation of Clinical Neurophysiology (IFCN) international guidelines (Rossini et al., 2015). In order to find the maximum amplitude of MEP that can stimulate FDI muscle, a figure‐of‐eight coil was positioned at a 45° angle relative to the sagittal line, and a single TMS stimulation was applied to the left primary motor cortex (M1) to identify the optimal stimulation site, which was then marked with a red indicator. After the position on M1 was determined, the output intensity was adjusted to achieve an average MEP amplitude of 1.0 ± 0.2 mV over 10 consecutive trials, then the output intensity is the motor threshold. The experiment commenced once the difference in mean amplitude between two consecutive MEPs was less than 20%. MEP amplitude is defined as the peak‐to‐peak amplitude, MEP latency was calculated as the time interval between the stimulus onset and the peak amplitude, both values derived from the average of 10 TMS trials with a 5‐s interstimulus interval.

The dependent variables were reported in terms of mean and standard deviation (SD). Normal distribution was confirmed through the Shapiro–Wilk test. A two‐way repeated measures analysis of variance was employed to assess the variance between acupuncture method and time effects. Bonferroni post hoc tests were utilized for specific comparisons. Statistical significance was established at *p* < .05. All statistics were performed using SPSS 26 software (IBM).

## 3 RESULTS

**FIGURE 2:**
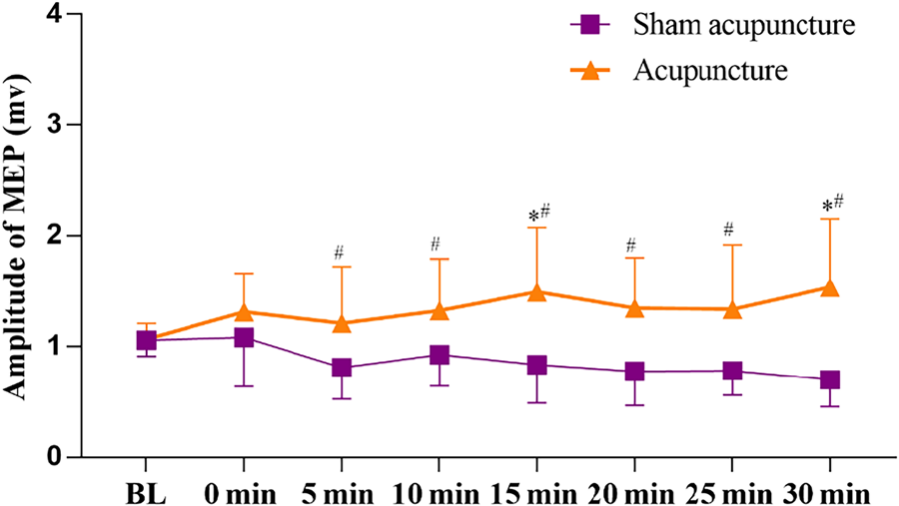
Comparison of the amplitude of motor evoked potential (MEP) between different acupuncture method and time. Values are means ± standard deviation (SD); significant difference was set at *p* < .05 (^*^, time effect; ^#^, acupuncture effect).

The findings indicated a significant interaction effect of MEP amplitude on acupuncture method and acupuncture time (*F*_(1, 38) _= 5.40, *p* < .001, *η* ^2 ^= 0.12). For the significant main effect of acupuncture method (*F*_(1, 38)_ = 39.17, *p* < .001, *η* ^2 ^= 0.51), the results indicated that the MEP amplitude from 5 min to 30 min were all significantly higher than baseline in acupuncture intervention group (*p* < .05). While no main effect was observed for acupuncture time, MEP amplitudes at 15 min (*p* = .007, MD = 0.43, 95% CI = 0.07–0.78) and 30 min (*p* = .002, MD = 0.47, 95% CI = 0.11–0.83) postacupuncture were significantly higher compared to baseline (Figure 2).

**FIGURE 3:**
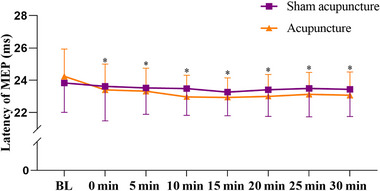
Comparison of the latency of motor evoked potential (MEP) between different acupuncture method and time. Values are means ± standard deviation (SD); significant difference was set at *p* < .05 (^*^, time effect; acupuncture effect).

With regard to latency of MEP, a significant interaction effect was also found (*F*_(1, 38) _= 3.78, *p* = .008, *η* ^2 ^= 0.09). Although no significant difference was observed in acupuncture method, a significant main effect was found for acupuncture time (*F*_(1, 38) _= 16.16, *p* < .001, *η* ^2 ^= 0.30), the results revealed that the latency of MEP from 0 min to 30 min were all significantly higher than baseline after acupuncture (*p* < .001; Figure 3).

**FIGURE 4:**
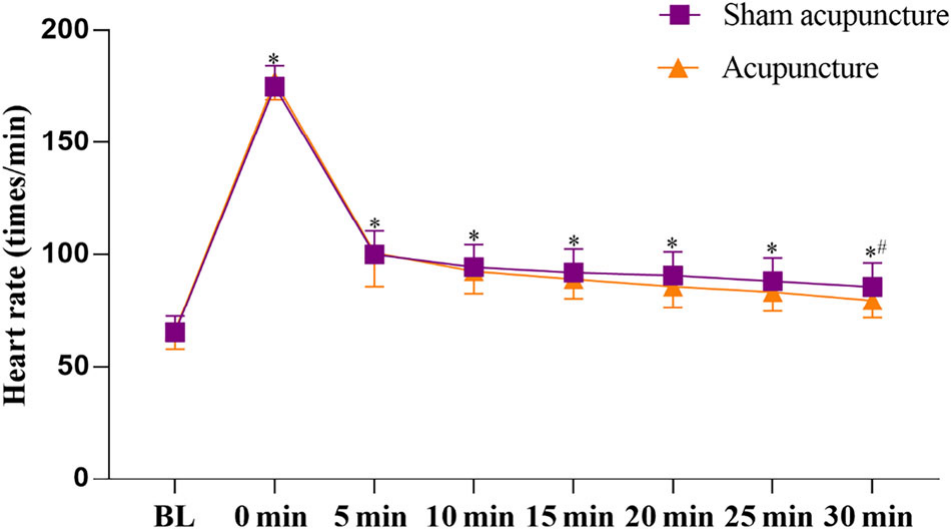
Comparison of the heart rate between different acupuncture method and time. Values are means ± standard deviation (SD); significant difference was set at *p* < .05 (^*^, time effect; ^#^, acupuncture effect).

The results of heart rate showed that there was interaction effect between the acupuncture method and acupuncture time (*F*_(1, 38) _= 2.85, *p* = .029, *η* ^2 ^= 0.07). Specifically, the acupuncture intervention group exhibited significantly lower heart rates compared to sham acupuncture after 30 min (*p* = .049, MD = −6.00, 95% CI = −11.97–0.03). For the significant effect of acupuncture time (*F*_(1, 38)_ = 1325.36, *p* < .001, *η* ^2 ^= 0.97), the results revealed that the heart rate from 0 min to 30 min was all significantly higher than baseline after acupuncture (*p* < .001; Figure 4).

**FIGURE 5:**
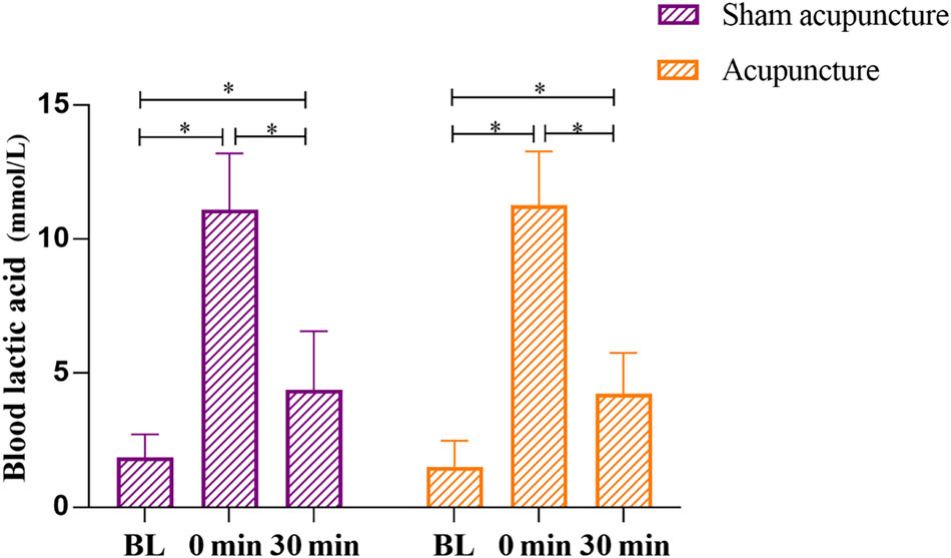
Comparison of the blood lactic acid (BLA) between different acupuncture method and time. Values are means ± standard deviation (SD); significant difference was set at *p* < .05 (^*^, time effect; acupuncture effect).

No significant interaction difference was found in BLA (*F*_(1, 38)_ = 0.378, *p* = .686, *η* ^2^ = 0.01). Further, although no significant difference was found in group effects, we found significant main difference on acupuncture time (*F*_(1, 38)_ = 529.11, *p* < .001, *η* ^2 ^= 0.93), the BLA in 0 min was significantly higher than baseline (*p* < .001, MD = 9.76, 95% CI = 8.69–10.83) and 30 min (*p* < .001, MD = −7.04, 95% CI = −8.10–5.97) in acupuncture intervention group (Figure 5).

## 4 DISCUSSION

The primary objective of our investigation was to assess the impact of acupuncture on exercise‐induced fatigue utilizing TMS. Our findings revealed that acupuncture has a beneficial influence on the restoration of motor cortex excitability and heart rate, particularly at the 30‐min postintervention.

Given to the fact that the alterations in excitability within the M1 region were found to be associated with the intensity of exercise. The fatigue model constructed in this experiment utilizing high‐intensity exercise to explore MEP amplitude after exercise intervention (Balbi et al., 2002). The results indicated a significant increase in MEP amplitude following acupuncture compared to sham acupuncture, along with a notable reduction in MEP latency postacupuncture compared to baseline levels. These findings are similar to results previously reported by Li et al. (2022), who demonstrated that electroacupuncture at back‐shu points enhanced motor cortex excitability and alleviated chronic fatigue. Also, this study supports those of other recent analyses reporting that acupuncture is an appropriate treatment for relieving fatigue and can improve the subjective rating of fatigue (Akimoto et al., 2003). The positive results after acupuncture intervention may be explained from metabolomics perspectives, compared with the relaxation after exercise, acupuncture can better regulate disturbances in the body, promote energy metabolism and choline metabolism, and improve reactive oxygen stress (Ma et al., 2015).

The study observed a notable decrease in heart rate following a 30‐min acupuncture intervention compared to a sham acupuncture intervention, the current data combined with previous data indicate that acupuncture has the potential to enhance heart rate during states of fatigue and facilitate the restoration of bodily functions (Jiang et al., 2019; Shu et al., 2016; Li et al., Li et al., 2005). Notably, recent evidence also suggests alternative therapies like Shiatsu, can generate positive effects on both mood status and cognitive performance from the metabolic and neuroendocrine levels (Lanza et al., 2018), leading to significant alleviation of fatigue.

BLA typically peaks 3–5‐min postexercise, often serving as a key indicator for assessing fatigue (Lucertini et al., 2017). However, our study did not track the immediate and 30‐min postexercise changes in BLA. A possible explanation for these results would be that blood sampling at the fingertip exerts an invasive stimulus on the subjects’ skin, which may produce pain sensation in the brain and affects the MEP during the experiment. Our results appear to contradict the findings presented by Poton and Polito (2016), who showed that high‐intensity exercise can lead to an increase in BLA following short‐term training, suggesting that brief training sessions may be more effective in elevating BLA levels compared to longer sessions (Kondoh et al., 1992). Further, Lin et al. observed lower BLA levels in the acupuncture group compared to the sham and control groups at 30‐min and 60‐min postacupuncture at ST 36 and PC 6 points (Lin et al., 2009, 2011). Xia and Wang (2016) demonstrated acupuncture could promote the significant reduction of BLA and creatine kinase in adolescent weightlifters with symptoms of lumbago. The discrepancies in these findings may be attributed to the use of a single intervention point and shorter retention time of acupuncture, potentially leading to an insignificant impact of acupuncture on BLA regulation.

There are several limitations to the present study that should be mentioned. First, due to insufficient time interval, it is hard to record cortical long‐interval cortical inhibition. Second, only a single intervention was conducted using ST 36 acupoints on exercise fatigue, which may contribute to the intervention intensity of acupuncture is not enough. Furthermore, this study only explored the influence of acupuncture on the M1, future studies could explore the mechanism of acupuncture on the brain utilizing functional near‐infrared spectroscopy and functional magnetic resonance imaging.

## 5 CONCLUSION

The results indicate that acupuncture intervention can improve the fatigue‐induced decrease in motor cortex excitability, promote the increase of MEP amplitude, shorten MEP latency, and restore heart rate. Therefore, acupuncture was suggested as a valuable intervention strategy for sports enthusiasts to alleviate fatigue and enhance sports performance.

## AUTHOR CONTRIBUTIONS

**Linghui Hu**: Conceptualization; data curation; formal analysis; investigation; methodology. **Zhen Wei**: Project administration; resources; software; supervision; validation. **Xiaolei Wang**: Visualization; writing—original draft; writing—review and editing. **Wei Wu**: Funding acquisition; supervision; validation; writing—review and editing.

## CONFLICTS OF INTEREST STATEMENT

The authors declare no conflicts of interest.

### PEER REVIEW

The peer review history for this article is available at https://publons.com/publon/10.1002/brb3.3575.

## Data Availability

The data are available from the corresponding author upon reasonable request.
